# How will artificial intelligence change medical training?

**DOI:** 10.1038/s43856-021-00003-5

**Published:** 2021-06-30

**Authors:** Shinjini Kundu

**Affiliations:** Department of Radiology, The Johns Hopkins Hospital, Baltimore, MD USA

**Keywords:** Preventive medicine, Diagnostic markers

## Abstract

Kundu discusses how artificial intelligence will transform medical practice and doctors’ training. The author explores the changing role of the clinician in the doctor-patient relationship, drawing parallels with the role of the pilot in light of increased automation in aviation.

## Teaser

Artificial intelligence is changing medicine and it will relieve physicians from the burden of rote knowledge. Here, I discuss how this might affect medical training, drawing from the example of how automation in aviation redefined the role of the pilot.

Technology inevitably shapes human behavior, and artificial intelligence (AI) is at the cusp of transforming medical practice. Continuous advances in monitoring health and disease have made medicine more precise, but they have also left doctors befuddled by mountains of healthcare data and ever-expanding medical knowledge that is becoming increasingly difficult to master and interpret. Medical experts sift through this vast amount of healthcare data to make diagnosis and treatment choices based on the most recent medical knowledge. The diagnosis and the treatment decisions are referred to as *data labels* in AI. From healthcare data labeled in this way, AI can learn the decision patterns to apply them to future cases on new data, unseen in the training set. AI is capable of generalizing and making decisions in unpredictable circumstances—in contrast to simple automation, which is good at exact routine tasks for which it has been designed but lacks the generalization capacity to make decisions in unforeseen contexts. Since the first medical device using AI was approved by the Food and Drug Administration (FDA) in 2016^1^, a rapid surge in the number of medical devices using AI has ensued; the number of publications using AI, including machine learning, in the life sciences increased >20-fold from 2010 to 2019^1^. AI can be applied to a wide range of healthcare activities, including treatment recommendations, patient monitoring, adherence checking, and medical record keeping. This is propelling clinical practice to an age where information and data are handled by machines, enabling post-knowledge physicians to focus on other things. Now that routine cognitive tasks can be assigned to machines, this is an opportunity to reconsider how medical schools should train doctors.

AI could alter the physician’s role in a similar way to how general automation altered the pilot’s role several decades ago.

In medicine, the need for “machine assistance” has undoubtedly never been greater. First, the doubling time of medical knowledge is merely 73 days today compared to 50 years in 1950^2^. Medical students would need to study >29 h every weekday to keep up with the primary care literature^3^. In fact, too much information drives clinical specialization. Today, 88% of internal medicine residents specialize, up from 7% in 1951–1960^4^. Second, as people live longer, they are more likely to develop multiple comorbidities, necessitating a tailored approach to treatment. Lastly, medical data in electronic medical records are growing at an unprecedented rate, in part due to the adoption of high-resolution imaging modalities, next-generation sequencing, and other technologies, as well as a wider array of tests and medications at clinicians’ disposal. No wonder freshly clad interns are seen with white coat pockets overflowing with flashcards, rounding notes, fishbone laboratories, and guides for drug dosing—the first year of residency is largely a training in the art of culling data.

One way to deal with this medical data and information explosion is to use AI to help make better sense of it. For example, there is already proof that AI can assimilate medical knowledge required for clinical thinking^5^. Last year, a company developed an AI system that outperformed doctors on a mock test of clinical reasoning^6^. The new Digital Health Applications Precertification Program of the FDA is also likely to speed up the transition of software as medical device to bedside^7^. In addition, tomorrow’s doctors may have access to a wider portfolio of assistive devices. Speech recognition is already automating clinical interview transcriptions. A smart electronic medical record may prompt a doctor to ask specific questions based on symptoms and might even suggest tests and diagnoses. While current prognostic calculators use only 5–10 variables, AI-based calculators could include substantially more, improving accuracy. Given more accurate disease assessment, smart tools could then recommend a menu of treatments considering patients’ allergies, current medications, and medical comorbidities. Dosage guidance could automatically account for patient weight, gender, and drug metabolism and excretion as relevant. Potentially, AI could help refine best practices by optimizing the scope and setting for newer treatment modalities, such as cancer immunotherapies. As AI becomes more widely used, knowledge and data may no longer differentiate the skill levels of physicians. As AI becomes part of the team and extends physicians’ skill sets, medical schools will need to emphasize a new set of competencies to keep up with how medical practice would evolve in the post-knowledge age.

In some ways, AI could alter the physician’s role in a similar way to how general automation altered the pilot’s role several decades ago. Initially, an aircraft was powered by a stick and rudder and the pilot’s intuition about how best to handle them was essential. Pilots learned basic psychomotor skills of keeping an aircraft suspended in flight, gauging the effect of multiple forces on a plane by gestalt^8^—so-called *stick-and-rudder flying*. However, as flying continually became more complex, with new components to control, automation enabled aircrafts to constantly adjust parameters, such as fuel efficiency, aviation, navigation, and communication with air controllers in real time through interacting automated systems^8^. Today, being a pilot entails seamlessly *switching* between stick-and-rudder skills and flight deck management as informed by system feedback. An effective mission relies on the *interaction* between the human and automated parts—not one or the other alone. Such automation brought clear advantages; simpler interfaces for humans, standardization, enhanced operational efficiency, and increased safety as nearly 80% of accidents were attributable to human error^8^. However, both too little and too much system feedback proved to be dangerous: too much could confuse the pilot and cause a crash, while too little may deter the pilot from acting on a system error. For example, the recent crashes of two Boeing 737 MAX aircraft just 4 months apart were caused by failure of a single malfunctioning sensor. Normally, planes rely on redundant systems to eliminate the risk of single point of failure, but the 737 MAX’s software relied on a single sensor despite having two. Pilots struggled to maneuver the plane in the absence of meaningful feedback, resulting in crashes^9^. This example should serve as a warning about the risks of using AI to make healthcare decisions, which should be factored in when building such a system.

Application of AI in healthcare comes with new risks. Overreliance on AI may reduce physicians’ situational awareness and create significant risks of being blindsided. Another risk of depending on AI is that, if it ceases to operate or is no longer capable of delivering the required services, there needs to be a safety valve—which means experts will still be needed. Furthermore, AI systems add another dimension beyond automation as they continuously learn from data. While AI programs could offer one of the biggest advantages by curating the best information through data-driven practices, they can also make errors on a large scale. Therefore, AI must always be a feedback-driven system, with users having a right to notify when AI-driven decisions are incorrect, so that the model can learn from its mistakes in subsequent iterations of training. There are also ethical considerations regarding the use of AI in healthcare. Physicians and patients often make trade-offs when deciding on treatments; one example is between quality of life and length of life. As a result, there is no such thing as a one-size-fits-all approach to patient treatment. It is important for AI systems to capture the complexity of multiple-choice scenarios, and when a medical decision necessitates a trade-off, it must still be delegated to the stakeholders.

Medical students play a pivotal role as they train with a plethora of new devices. Keeping patients at the center of the mission, doctors-in-training could learn how to manage patient data more like managing the signs on a flight deck—exploring the impact of multiple influences on patient health, such as social determinants, clinical diagnosis and care, timely decisions, and teamwork with other health professionals^8^. In post-knowledge medicine, the focus of training might shift from biology to psychology and sociology, focusing on empathy and a greater understanding of socioeconomic structures. Knowledge-centered or even therapeutic interaction has a smaller impact on wellbeing than is commonly assumed. According to some studies, symptom and knowledge-centered treatments have just 10–15% impact on health^10^, while a combination of social determinants makes the biggest difference in results^11^. Only when physicians are better equipped to consider the various data components of a patient’s health and to resolve hidden barriers to health, such as lack of access to medicine, transportation, adequate nutrition, and undiagnosed complex diseases, will they be able to concentrate on long-term patient well-being. This is only possible if a physician can work interactively with AI to develop a treatment plan that is specifically tailored for a patient’s needs. Further, medical education must teach students how to scrutinize and crosscheck knowledge and data, as well as recognize when they must fall back on stick-and-rudder medical skills, where to find the experts, and when to seek collaboration with other members of healthcare team to address the hidden barriers to health.

Finally, some could argue that, as the physician’s role changes to that of a patient healing supervisor, doctors will become less happy in their jobs. In aviation, pilots sometimes became bored and disengaged while serving as flight deck supervisors^8^. “Human-centered automation,” which compensates for human operators’ shortcomings via visual assistance and alerts^8^, provided a solution that effectively engaged pilots and enriched their skill set. Remembering the human at the center is a keystone opportunity to return medicine to its original ethos. Today, intern physicians strike a frenzied balance between administrative work, education, charting, and other activities, leaving as little as 12% of time for direct patient care^12^. Yet, each year, thousands of newly minted medical students recite the Hippocratic Oath from one of the oldest texts in history. The hallowed bond between the ill and healing practitioner has remained steadfast, defining the heart of the profession. When physicians give time to the patient–doctor relationship, outcomes improve^13^. If AI can relieve physicians of duties competing for physician time, then post-knowledge medicine will return physicians to the bedside—where the sacred relationship between an ill patient and a compassionate physician started.
